# Aldo Félix Craievich (1939–2023)

**DOI:** 10.1107/S1600577523005416

**Published:** 2023-06-21

**Authors:** Diego G. Lamas

**Affiliations:** a Instituto de Tecnologías Emergentes y Ciencias Aplicadas (ITECA), CONICET-UNSAM, Escuela de Ciencia y Tecnología, Laboratorio de Cristalografía Aplicada, Campus Miguelete, Edificio Labocluster, Av. 25 de mayo 1169, (1650) San Martín, Provincia de Buenos Aires, Argentina

**Keywords:** obituary, Aldo Félix Craievich

## Abstract

Obituary for Aldo Félix Craievich.

On 24 April 2023, we received the very sad news of the passing of Professor Dr Aldo Félix Craievich at the age of 84. He was a brilliant Argentine–Brazilian crystallographer who not only had the remarkable vision to create the first synchrotron in the southern hemisphere but also played a leading role in the construction of the Brazilian Synchrotron Light Laboratory (LNLS) in Campinas, São Paulo, Brazil, serving as its first deputy director and head of the scientific department. He coordinated the projects of the LNLS beamlines and the construction of the first seven of them, but also he put in a lot of effort to create the user community of LNLS. He organized and gave by himself numerous courses and conferences on synchrotron light techniques throughout Latin America, becoming a mentor and great teacher for many crystallographers in the region.

[Chem scheme1] 
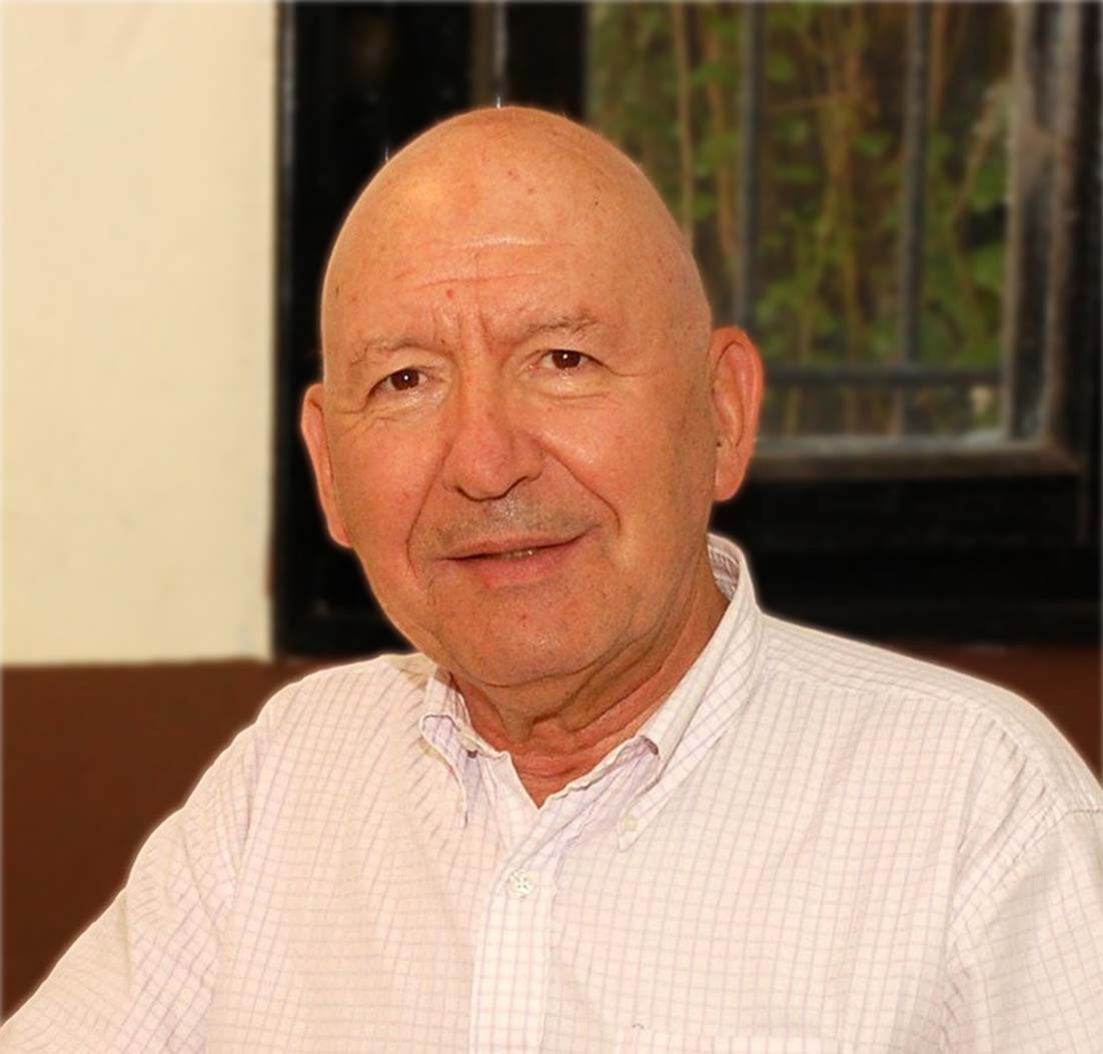


Aldo was born in 1939 in the city of Zavalla, in the Province of Santa Fe, Argentina, and lived in this country until age 34, except for when he resided in France to conduct the research work for his PhD thesis. He graduated in Physics in 1964 at the Balseiro Institute of the National University of Cuyo (San Carlos de Bariloche, in the Argentine Patagonia), where he received a highly advanced education that profoundly impacted his future career as a researcher. In 1965, he joined the Institute of Mathematics, Astronomy and Physics (IMAF, currently FaMAF) of the National University of Córdoba (UNC, Córdoba City), where he was tasked with creating a laboratory of X-ray techniques. To gain a deeper understanding of these techniques and complete his training in experimental physics, he decided to undertake a PhD. In mid-1966, he moved to the Laboratoire de Physique des Solides (LPS, Laboratory of Solid-State Physics) in Orsay, France, which at that time was part of the University of Paris and later became part of the newly established University Paris-Sud or University Paris XI (nowadays University Paris-Saclay). Thanks to the support of the authorities at IMAF and of another Argentine who had worked there, Alberto Bonfiglioli, Aldo was able to join the research group of the renowned crystallographer André Guinier, widely known for his contributions to small-angle X-ray scattering (SAXS) among others. Aldo presented his PhD thesis at the Balseiro Institute in early 1969, and from that moment on he resumed the establishment of the X-ray laboratory at IMAF, installing X-ray diffraction and SAXS instruments. In the case of the latter, this was the second one in the country, following the facility implemented by Bonfiglioli at the Constituyentes Atomic Center of the National Atomic Energy Commission (CAC-CNEA) in Buenos Aires. Unfortunately, both SAXS facilities were discontinued some years later when both researchers left their institutions, and Argentina did not have similar instruments for decades. This situation only started to change a few years ago, largely thanks to Aldo’s work in teaching and promoting X-ray techniques and crystallography.

Aldo worked at IMAF for a few years and published the first papers using the equipment from the laboratory he established. However, in 1973 he accepted an invitation from Professor Yvonne Mascarenhas to work at the Institute of Physics and Chemistry of São Carlos (IFQSC, now IFSC) in São Carlos, São Paulo State, Brazil. There, he set up the first SAXS instrument in Brazil, making again a valuable contribution to the region. He initially planned to stay at IFQSC for only one year, but, because of the serious political situation in Argentina, he extended his stay, and years later, when the Argentine democracy was restored, he led the very important project to create a synchrotron facility. As a result, Aldo resided in Brazil for the rest of his life. In relation to this, it is worth mentioning that Aldo never forgot his home country: he was always grateful for the education he received and returned many times to visit family, colleagues, teachers and friends. Furthermore, he made significant contributions to the progress of science in Argentina.

At IFQSC, Aldo continued his previous work on glassy materials using SAXS experiments, but after a few years he decided to align his research work with the institute’s trend of studying biological systems and started by exploring organic molecular crystals. To deepen his knowledge in this area, he embarked on a postdoctoral stay at the LPS in 1976, returning to IFQSC the following year to initiate this new line of research, which also relied on SAXS experiments.

In 1980, Aldo decided to leave São Carlos to undertake a new challenge in Rio de Janeiro: the creation of an X-ray laboratory at the Brazilian Center for Research in Physics (CBPF), an institution that had a reputation for excellence in theoretical research. Since he had to wait approximately a year for the purchase of the instruments, he decided to do another stay in Orsay, but this time at the recently established Laboratoire pour l’Utilization du Rayonnement Electromagnétique (LURE, Laboratory for the Use of Electromagnetic Radiation). At that time, synchrotron radiation sources were still in their early development stages, and the LURE laboratory, being one of the first facilities open to users, played a crucial role in expanding the use of techniques based on this radiation. During Aldo’s stay at LURE from 1981 to 1982, which was aimed primarily at conducting *in situ* SAXS experiments on glassy materials and exploring extended X-ray absorption fine-structure (EXAFS) spectroscopy, the idea of creating the first synchrotron radiation source in Brazil emerged. This initiative gained strength when Aldo organized a visit to LURE by the president of the National Council for Scientific and Technological Development (CNPq) of Brazil in early 1982. Shortly after, the Synchrotron Radiation Project (PRS) of CNPq was established. This was developed from 1982 to 1985, with Aldo serving as the director of its executive committee from 1983. This role led him to promote the project and the importance of synchrotron radiation techniques in Brazil and other countries in Latin America. Additionally, Aldo coordinated a CNPq scholarship program for human resource development in these techniques. Finally, in 1985, it was decided to create the Brazilian Synchrotron Light Laboratory (LNLS) in Campinas, São Paulo State, and in 1986 CNPq designated Cylon Gonçalves da Silva, a professor at the Institute of Physics of the University of Campinas, as the director of LNLS, while Aldo served as the deputy director.

The construction of LNLS took ten years and had unique characteristics. Particularly noteworthy is the fact that most of the components needed for the first source, called UVX, and the beamlines were manufactured either at LNLS itself or by Brazilian industry. Undoubtedly, this project can be considered one of the most important milestones in science and technology in Latin America. While the UVX source construction project was led by Antonio Ricardo D. Rodrigues, Aldo took charge of leading the projects for the beamlines and the construction of the first seven, which were made available to users in July 1997. Additionally, Aldo recognized the vital importance of building a user community, the true ‘owners’ of an open facility like LNLS, and, to achieve this, he taught courses and lectures not only in Brazil but also in many other Latin American countries, including Argentina, Chile, Colombia, Cuba, Mexico, Peru, Uruguay and Venezuela. In 1989, long before the laboratory was opened to users, he organized the First LNLS User Meeting, an activity that has continued every year and still takes place with increasing interest.

It is worth noting that Aldo believed that in order to successfully fulfill his important role at LNLS he needed to stay updated with advances in synchrotron radiation instrumentation and techniques. Therefore, every year he returned to the LURE synchrotron as a user for at least one or two weeks. He continued conducting experimental work, particularly in the study of nanomaterials and glassy materials using SAXS techniques, and publishing the results of his high-level scientific research work.

Towards the end of 1997, after many years of intense work and with LNLS operating successfully, Aldo decided to leave the institution and dedicate himself fully to research and university teaching. Therefore, in January 1998, he joined the Institute of Physics at the University of São Paulo (IF-USP) in São Paulo city, where he obtained a position as a full-time professor. However, he never distanced himself from LNLS. He not only remained a frequent user but also continued to work on building the user community. Aldo continued to deliver courses and lectures, and promoted scientific collaborations with groups sharing similar research interests. It was during this time that I had the honor of collaborating closely with Aldo, sharing 20 years of scientific challenges, teaching activities, crystallography outreach and friendship.

In 2009, upon reaching the age of 70, Aldo was forced to retire, as mandated by Brazilian law. However, he continued as a senior professor at IF-USP until the end of his days. He never stopped travelling the world to teach and learn, always with great enthusiasm. He followed with great interest the creation of LNLS’s second source, Sirius, whose characteristics were discussed with users in the same year of Aldo’s retirement. Construction began in 2013, and the first users were welcomed in 2020. Much earlier than its operational start-up, it was understood that Sirius would be one of the most advanced synchrotron sources in the world, a fourth-generation source, and that the user community needed to prepare itself to match such advancements. Aldo accompanied us in this process and encouraged us to continue growing as researchers. The COVID-19 pandemic and subsequent health issues prevented him from further travels, but he remained engaged in all LNLS activities and the numerous scientific societies that interested him, including the International Union of Crystallography (IUCr), the Brazilian Crystallographic Association (ABCr), the Argentine Crystallographic Association (AACr), the Latin American Crystallographic Association (LACA), the Brazilian Society for Materials Research (SBPMat), the Argentine Association of Materials (SAM) and the Argentine Physical Association (AFA), among others. Regarding the IUCr, it is worth mentioning that Aldo served as co-editor of the *Journal of Synchrotron Radiation* from 2009 to 2020.

Aldo received numerous awards and distinctions throughout his career. In 1980, he became a full member of the São Paulo Academy of Sciences (Brazil) and, since 2015, also of the Brazilian Academy of Sciences. He received recognition from the LNLS (staff and users), ABCr, AACr, SBPMat, the National University of the Littoral and the Balseiro Institute. Additionally, he received the Mercosur Science and Technology Prize twice (2004 and 2010), jointly with colleagues from Argentina and Brazil, in the ‘integration’ category, awarded by UNESCO and the Network of Science and Technology of Mercosur (RECyT). ▸

Working with Aldo was a fascinating experience, not only for me but also for many other colleagues. Whenever there was something we didn’t understand, Aldo would take a pencil and start reasoning deeply, not letting go of the subject until the issue was resolved. He always encouraged us to comprehend the phenomena we were studying in great detail, believing that any question could be solved with enough effort. By sharing scientific activities with him, we discovered that we had so much more to offer as scientists if we did not settle for superficial answers or quick solutions. Another aspect I want to highlight is that Aldo was incredibly generous and open with his knowledge, a quality that unfortunately is not always found and one we truly appreciated. Furthermore, Aldo was a person of great culture, and when he shared anecdotes of his experiences he always left us with valuable lessons.

The first LNLS source, UVX, ceased operation in 2019 after 22 years. However, Sirius is now fully operational, and the construction of new beamlines shows a bright future ahead. In addition, after 40 years since the initiation of the PRS-CNPq project, the large Latin American user community, which was created through Aldo’s generous teaching efforts, is now embarking on new challenges in the development of Sirius. The renovated synchrotron facility at LNLS and its well established user community are the main legacies of Professor Dr Aldo Félix Craievich, who had the vision to recognize the tremendous importance of creating the first multi-user synchrotron radiation laboratory in the southern hemisphere and dedicated most of his scientific life to turning that vision into a reality. At the same time, he promoted the fundamentals of crystallography and many of its techniques. In recent years, his SAXS courses in Argentina led to the establishment of two advanced instruments dedicated to this technique, both of them being opened to the scientific community. Following Aldo’s example, the leading researchers of these SAXS facilities continue to disseminate the fundamentals and applications of the technique he learned from Guinier. We will deeply miss Aldo, our dear friend, teacher and mentor.

## Figures and Tables

**Figure 1:**
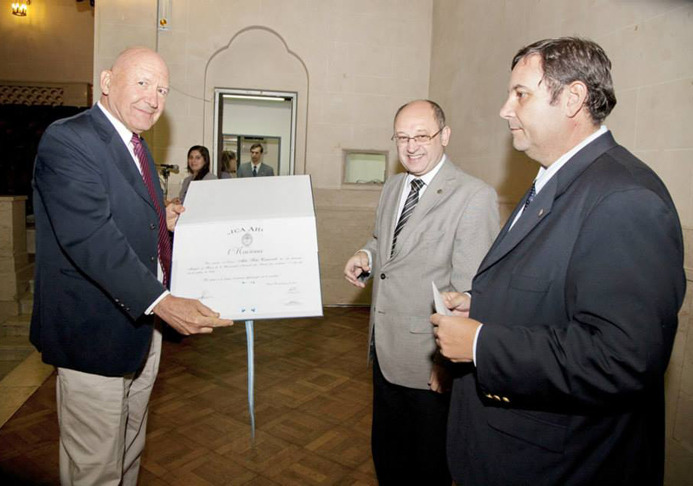
Photograph taken during the VIII Annual Meeting of the Argentinian Association of Crystallography, in Santa Fe City, in 2012. Professor Dr Aldo Félix Craievich was recognized as a guest of honor of the National University of the Littoral.

